# Higher fish but lower micronutrient intakes: Temporal changes in fish consumption from capture fisheries and aquaculture in Bangladesh

**DOI:** 10.1371/journal.pone.0175098

**Published:** 2017-04-06

**Authors:** Jessica R. Bogard, Sami Farook, Geoffrey C. Marks, Jillian Waid, Ben Belton, Masum Ali, Kazi Toufique, Abdulla Mamun, Shakuntala H. Thilsted

**Affiliations:** 1 School of Public Health, The University of Queensland, Herston, Queensland, Australia; 2 Agriculture Flagship, Commonwealth Scientific and Industrial Research Organisation (CSIRO), St Lucia, Queensland, Australia; 3 Bangladesh Institute of Development Studies, Sher-e- Bangla Nagar, Dhaka, Bangladesh; 4 Helen Keller International, House 10E, Gulshan 2, Dhaka, Bangladesh; 5 Institute of Public Health, Heidelberg University, Heidelberg, Germany; 6 Department of Agricultural, Food & Resource Economics, Michigan State University, East Lansing, Michigan, United States of America; 7 WorldFish, Batu Maung, Bayan Lepas, Penang, Malaysia; Swinburne University of Technology, AUSTRALIA

## Abstract

Malnutrition is one of the biggest challenges of the 21^st^ century, with one in three people in the world malnourished, combined with poor diets being the leading cause of the global burden of disease. Fish is an under-recognised and undervalued source of micronutrients, which could play a more significant role in addressing this global challenge. With rising pressures on capture fisheries, demand is increasingly being met from aquaculture. However, aquaculture systems are designed to maximise productivity, with little consideration for nutritional quality of fish produced. A global shift away from diverse capture species towards consumption of few farmed species, has implications for diet quality that are yet to be fully explored. Bangladesh provides a useful case study of this transition, as fish is the most important animal-source food in diets, and is increasingly supplied from aquaculture. We conducted a temporal analysis of fish consumption and nutrient intakes from fish in Bangladesh, using nationally representative household expenditure surveys from 1991, 2000 and 2010 (n = 25,425 households), combined with detailed species-level nutrient composition data. Fish consumption increased by 30% from 1991–2010. Consumption of non-farmed species declined by 33% over this period, compensated (in terms of quantity) by large increases in consumption of farmed species. Despite increased total fish consumption, there were significant decreases in iron and calcium intakes from fish (P<0.01); and no significant change in intakes of zinc, vitamin A and vitamin B12 from fish, reflecting lower overall nutritional quality of fish available for consumption over time. Our results challenge the conventional narrative that increases in food supply lead to improvements in diet and nutrition. As aquaculture becomes an increasingly important food source, it must embrace a nutrition-sensitive approach, moving beyond maximising productivity to also consider nutritional quality. Doing so will optimise the complementary role that aquaculture and capture fisheries play in improving nutrition and health.

## Introduction

Malnutrition and poor diet are the leading causes of the global burden of disease, with nearly 800 million people suffering from hunger and two billion people suffering from micronutrient deficiencies [1]. Undernutrition alone accounts for 45% of all child deaths, and prevents millions from reaching their developmental potential, with profound social and economic impacts [2]. The immensity and urgency of this global challenge is reflected in the Unites Nations Sustainable Development Goals (SDGs), with goal two specifically aiming to end all forms of malnutrition [1].

Bangladesh experiences amongst the worst malnutrition rates in the world. The most recent estimates show that 36% of children under 5 years are stunted, 33% of children under 5 are underweight, 19% of adult women are undernourished [3], and millions live with various micronutrient deficiencies [4]. It is estimated that this costs Bangladesh USD 1 billion each year in economic productivity forgone [5].

Fish is an essential component of diets around the world, providing more than 3 billion people with around 20% of their animal-source protein [6], and an even greater contribution in many developing countries [7]. In Bangladesh, fish is by far the most important nutrient-rich food in the diet, across all population groups; urban and rural, rich and poor, female and male, young and old [8, 9]. However, the contribution of fish to food and nutrition security is often overlooked [6, 10] and even when acknowledged, is typically only defined in terms of animal protein [11]. However, fish is a rich source of multiple nutrients: essential fats, vitamins and minerals of high bioavailability, which all play critical roles in human health, growth and development, cognition and disease prevention [12–15]. Another rarely recognised nutritional characteristic of fish is that micronutrient content varies widely by species. Common farmed species are generally of lower nutritional quality compared to non-farmed species harvested from capture fisheries, as shown by analyses from Bangladesh [16].

Global fish supply is undergoing a profound transition. World supply of fish doubled from an average of 9.9 kg/capita/year during the 1960s to 20.1 kg/capita/year, in 2014, and aquaculture now provides more than half of the global supply of fish available for human consumption [11]. On the other hand, global capture fisheries production peaked in the 1990s and has plateaued since [11]. Other estimates show production peaked at a much higher level and has since been in more rapid decline [17]. As a result, it is projected that future global fish demand growth will be met entirely from aquaculture [18].

This global transition is mirrored at the national level in Bangladesh where per capita fish supply increased from 7.6 kg/capita/year in 1990 to 19.2 kg/capita/year in 2013 [19]. Over this same period, the share of aquaculture in Bangladesh’s fish supply increased from 23% to 55% and the quantity of farmed fish produced grew 810%, from 0.2 million tonnes to 1.96 million tonnes [20, 21]. Bangladesh is now the world’s sixth largest producer of aquaculture products [11].

The scale of this transition has given rise to concerns over the implications for nutrient supply and associated nutrition outcomes [22, 23], but its effects have never been examined empirically. Given how many people depend on fish as a food source and are also at risk of malnutrition, this is an important omission in the literature. This leads us to pose the question, how has the transition from capture fisheries to aquaculture affected micronutrient intakes in Bangladesh?

To answer this, we combine detailed fish consumption data from nationally representative household income and expenditure surveys (HIES) over a 20 year time series from 1991 to 2010, with comprehensive species-specific nutrient composition data for fish. We hypothesize that, either: 1) gains in farmed fish intakes should have raised micronutrient intakes from fish in Bangladesh, or; 2) the inferior nutritional quality of farmed fish has been insufficient to offset declines in nutrient intakes from capture fisheries. Both possibilities have important policy and health implications for Bangladesh and other developing countries where fish makes an important contribution to diets.

The rest of the paper is set out as follows: Firstly, we describe trends in apparent fish consumption patterns from 1991 to 2010. We also present the proportion of surveyed households consuming other animal-source foods (ASFs) in each survey year to give context as to the relative importance of fish in the diet. We then estimate changes in nutrient intakes from fish over time. The key micronutrients of interest are: iron and zinc, both considered ‘problem nutrients’ in developing countries [24]; calcium, as fish is known to be an important dietary source in Bangladesh [25]; and vitamin A and vitamin B12; as deficiency of both these micronutrients is widespread in Bangladesh [4]. We also examine results within extreme poor, moderate poor and non-poor households, and rural and urban areas to further understand the importance of nutrient intake from fish among different socioeconomic groups. We conclude by showing how these results contribute to understanding of the links between capture fisheries, aquaculture and nutrition, particularly for vulnerable groups, and advance recommendations for mitigating the impacts of this transition on human nutrition and health in Bangladesh and the developing world.

## Methods

### Household surveys

Nationally representative household food consumption data from the Household Expenditure Survey 1991, and HIES 2000 and 2010 in Bangladesh were used to estimate apparent fish consumption [26–28]. The three independent cross-sectional survey sample designs were based on a two-stage stratified sampling technique; primary sampling units were selected with probability proportional to size in the first stage, and households were selected by systematic random or circular sampling in the second stage [29–31]. General characteristics of surveyed households are described in Table 1. These are consistent with broader demographic trends in Bangladesh, including increasing urbanisation, decreased poverty rates, reduced fertility rates and an aging population, along with improved access to water and sanitation, and health care [3]. Of note is the considerable reduction in the proportion of extreme poor households between 1991 (40%) and 2010 (17%).

**Table 1:** Characteristics of surveyed households 1991–2010.

Characteristic	1991	2000	2010
Total number of HH	5,745	7,440	12,240
Rural HH, n (% of total)	4,971 (87%)	5,932 (80%)	8,956 (73%)
HH size, mean (SD)	5.35 (2.54)	5.20 (2.23)	4.50 (1.86)
AMEs per HH, mean (SD)	4.11 (2.10)	3.98 (1.81)	3.53 (1.53)
**Age group (year)**			
<2 (%)	4.2	4.1	3.6
2–5 (%)	13.4	10.2	8.4
6–14 (%)	26.1	25.4	22.7
15–49 (%)	45.9	47.9	50.9
>50 (%)	10.4	12.4	14.4
**Poverty group**			
Extreme poor (%)	40.0	35.9	16.9
Moderate poor (%)	15.0	13.0	12.6
Non-poor (%)	45.0	51.1	70.5

HH, household; SD, standard deviation; AME, adult male equivalent.

Means adjusted using sample weights.

Food consumption data were collected by trained interviewers using one-day recall for the 1991 survey over a period of 30 days, and using two-day recall for the 2000 and 2010 surveys to obtain food consumption over 14 consecutive days. Data collection was structured throughout the year, thereby controlling for seasonal effects.

Poverty status was defined, using the cost of basic needs method as per survey reports, with households categorised as extreme poor, moderate poor and non-poor. The food poverty line (FPL) was estimated as the cost of a basic food basket that meets the energy needs of an adult, and the non-food poverty line (NFPL) was estimated as the cost of non-food expenditure by households close to the FPL. Thresholds for each line were set for each survey year for each district and for rural and urban areas by the Bangladesh Bureau of Statistics (BBS). Extreme poor households are those with total expenditures at or below the FPL, and the moderate poor households are those with total expenditures at or below the NFPL and above the FPL.

Fish species recorded in the survey were grouped according to their dominant production sector; either capture fisheries (non-farmed) or aquaculture (farmed), for each survey year, allowing comparison of the relative contribution that each sector makes to fish consumption over time (see Detailed methods and Table A in S1 File). Results are presented per Adult Male Equivalent (AME). AME reflects the energy requirements of individual households members, based on age and sex, as a proportion of an adult male, providing a more accurate estimate of the adequacy of household food consumption compared to per capita intake [32] (see Detailed methods and Table B in S1 File). Households with unrealistic levels of fish consumption in the local context (>500 g/AME/day, for comparison, mean fish consumption was 54 g/AME/day) were excluded (n = 15 in 1991, from a total of 5,745 households). Data on the quantity of each fish species consumed were then combined with species level nutrient composition data to estimate apparent nutrient intakes from fish, at each time point [16, 33]. Fish species consumption is recorded in the surveys according to common Bangla names, which may represent several distinct species. In these cases, the average nutrient composition of several applicable species was used (see Table B in S1 File). For a small number of fish species recorded in the surveys, data were not available for vitamin A content (4–6 species across the three surveys, see Table B in S1 File for details) or vitamin B12 content (7–13 species across the three surveys). A small number of households consuming only fish with these missing data were therefore excluded from analysis of those specific nutrients (vitamin A, n = 171; vitamin B12, n = 465) to minimise impacting the results. The proportion of households consuming some quantity of fish, eggs, poultry, meat or dairy within the survey period (compared to total households), is also reported. This data is used to reflect consumption patterns of other ASFs relative to fish, over time.

### Statistical analysis

All statistical analyses were conducted using STATA (version 12.1, StataCorp, College Station, TX, USA). Regression analyses were used to estimate mean fish consumption and mean nutrient intakes from fish, at each time point (P<0.01, using sample weights provided by BBS and adjusting for clustering of primary sampling units in survey design). All primary outcome variables (fish consumption and nutrient intakes from fish, per AME/day) were positively skewed in distribution and log transformation did not produce a Normal distribution. Non-parametric tests and equality of means were not appropriate, given the need to apply sample weights. However, a sensitivity analysis was conducted, using quantile regression which is suitable for non-parametric analyses and is also not sensitive to the presence of outliers [34]. This analysis revealed similar trends and statistical significance; any deviations to main results are explained in footnotes to results tables. Unfortunately, quantile regression in STATA is not able to adjust for both clustering in survey design and survey weights simultaneously, and so, the sensitivity analyses presented here is based on quantile regression, adjusting for survey weights only. Overall strengths and limitations of the analysis are detailed in the S1 File.

## Results

### Fish consumption

Analysis of total fish consumption nationally shows no significant change between 1991 and 2000, followed by an increase from 53.7 g/AME/day in 2000 to 68.2 g/AME/day in 2010 (Table 2). However, examining consumption with respect to poverty and location groups over this period shows disparate trends. Between 1991 and 2000, there were no significant changes in fish consumption among all poverty groups and rural households, but a significant decrease among urban households. From 2000 to 2010, fish consumption increased significantly among urban, rural and non-poor households, and increased slightly but not significantly among extreme and moderate poor households. Overall, national mean fish consumption increased by 30% between 1991 and 2010. The *relative* increase between 1991 and 2010 was greatest among extreme poor households (19%, given their much lower intakes in 1991 compared to other poverty groups); however as expected, mean fish consumption was consistently much higher among non-poor households compared to moderate and extreme poor households.

**Table 2:** Mean total, non-farmed and farmed fish consumption (g/AME/day, % of total fish) over time.

	1991		2000		2010		Change 1991–2010	
	Mean [% of total fish]	SE	Mean [% of total fish]	SE	Mean [% of total fish]	SE	g/AME/day	% ‡
	**Total fish**							
National	52.5	1.9	53.7§	1.3	68.2*	1.2	+15.7†	30
**Location**								
Rural	49.5	2.2	53.8§	1.5	63.9*	1.3	+14.5†	29
Urban	72.0	3.1	53.5*	2.5	80.0††	1.9	+8.0†	11
**Poverty group**								
Extreme poor	33.7	1.8	37.2§	1.2	40.0§	1.1	+6.3†	19
Moderate poor	51.5	2.2	51.1	1.8	53.2§	1.3	+1.7§	3
Non-poor	69.6	2.5	66.0	1.5	77.7*	1.2	+8.1†	12
	**Non-farmed fish**							
National	50.4 [96]	1.9	37.6* [70]	1.2	33.8* [49]	0.9	-16.6†	-33
**Location**								
Rural	47.4 [96]	2.1	37.5* [70]	1.4	30.6* [48]	1.0	-16.8†	-35
Urban	69.4 [96]	3.1	37.8* [71]	2.0	42.2§ [53]	1.6	-27.2†	-39
**Poverty status**								
Extreme poor	32.6 [97]	1.7	27.8** [75]	1.1	21.3* [53]	0.7	-11.3†	-35
Moderate poor	49.9 [97]	2.2	37.9* [74]	1.8	25.7* [48]	0.9	-24.2†	-49
Non-poor	66.4 [95]	2.5	44.4* [67]	1.4	38.2* [49]	1.0	-28.2†	-43
	**Farmed fish**							
National	2.1 [4]	0.2	16.1* [30]	0.6	34.5* [51]	0.7	+32.4†	-
**Location**								
Rural	2.0 [4]	0.3	16.2* [30]	0.7	33.3* [52]	0.8	+31.3†	-
Urban	2.6 [4]	0.3	15.7* [29]	1.2	37.7* [47]	1.1	+35.2†	-
**Poverty status**								
Extreme poor	1.1 [3]	0.2	9.4* [25]	0.5	18.7* [47]	0.7	+17.6†	-
Moderate poor	1.6 [3]	0.3	13.2* [26]	0.7	27.5* [52]	0.9	+25.9†	-
Non-poor	3.2 [5]	0.4	21.6* [33]	0.8	39.5* [51]	0.7	+36.4†	-

SE, standard error; AME, adult male equivalent.

Means adjusted for clustering and sample weights. n = 25,425. Significance of sensitivity analysis only noted when different from main analysis.

* Significantly different from previous survey year at P<0.01.

** Significantly different from previous survey year at P<0.05.

† 2010 mean is significantly different from 1991 mean at P<0.01.

†† 2010 mean is significantly different from 1991 mean at P<0.05.

‡ Change (%) not calculated when baseline in 1991 was less than 5 g/AME/day.

§ In sensitivity analysis, median intake was significantly different than median intake in previous year at P<0.05.

Examining fish consumption over time according to source of production (non-farmed or farmed) demonstrates contrasting trends (see Table 2). Consumption of non-farmed fish decreased significantly, both nationally (-33%) and across all poverty and location groups between 1991 and 2010, whereas consumption of farmed fish increased significantly for all poverty and location groups, from a national average intake of 2.1 g/AME/day in 1991 to 34.5 g/AME/day in 2010. Farmed fish made up a larger proportion of total fish consumption for the non-poor (5–51%) compared to the extreme poor (3–47%), consistently over time.

### Consumption of fish and other animal-source foods

The importance of fish compared to other ASFs in diets over time is shown in Table 3. Nearly all households in each survey year (95–99%) reported consumption of fish in the survey recall period, compared to much more variable consumption of other ASFs; 36–87% reported egg consumption, 8–59% reported meat consumption, 9–59% reported poultry consumption and 23–68% reported dairy consumption; within the survey recall period. As expected, extreme poor and rural households were much less likely to have consumed all ASFs within the survey recall period, compared to non-poor and urban households, respectively, in each survey year. These trends are in line with data reported elsewhere, with fish being by far the most important ASF in diets of women [35].

**Table 3:** Proportion of total households (%) reporting consumption of selected animal-source foods in the survey period*.

	Fish			Eggs			Meat			Poultry			Dairy products		
	1991	2000	2010	1991	2000	2010	1991	2000	2010	1991	2000	2010	1991	2000	2010
National	96.8	98.4	98.7	49.5	63.8	73.9	30.1	39.7	26.4	18.6	24.3	43.2	43.6	53.1	50.8
**Location**															
Rural	96.5	98.2	98.4	48.4	60.9	69.1	27.2	34.9	21.1	18.2	21.6	37.6	42.1	50.0	47.5
Urban	99.2	99.1	99.3	56.6	75.4	86.9	49.3	58.6	40.8	21.8	34.6	58.5	53.2	65.1	59.9
**Poverty status**															
Extreme poor	94.6	96.9	96.2	36.0	49.3	53.6	17.0	23.5	8.1	8.6	10.8	18.4	26.9	33.4	23.4
Moderate poor	97.6	98.7	98.7	51.4	62.6	68.9	27.4	34.2	18.0	18.3	19.4	30.8	43.4	49.8	38.3
Non-poor	98.6	99.4	99.2	60.9	74.4	79.6	42.8	52.5	32.3	27.7	35.0	51.4	58.6	67.7	59.7

* Note that the recall period for the 1991 survey is 30 days compared to 14 days in 2000 and 2010.

N = 25,425. Proportions adjusted using sample weights.

### Nutrient intakes from fish

**Fig 1:**
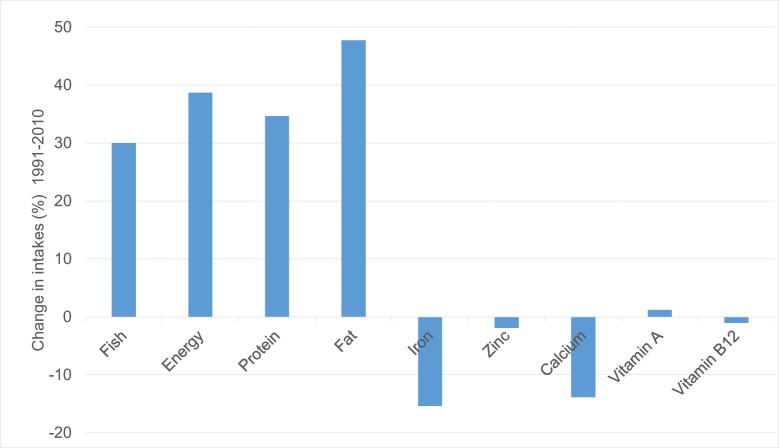
Change (%) in fish consumption and nutrient intakes from fish from 1991 as baseline to 2010*. *All changes except for zinc, vitamin A and vitamin B12 between 1991 and 2010 are statistically significant P<0.01.

There were significant increases in average energy, protein and fat intake from fish, in line with increasing total fish consumption, both nationally and for all poverty groups between 1991 and 2010 (P<0.01, increase in energy intake among moderate poor households significant at P<0.05, Table 4), except for protein intake among moderate poor households in which there was no significant change. However, there was no significant change in average intakes of zinc, vitamin A and vitamin B12; and consumption of iron and calcium significantly decreased (P<0.01), despite a national increase of 30% in the total quantity of fish consumed (proportional changes shown in Fig 1).

**Table 4:** Mean nutrient intakes (nutrient/AME/day) from fish, nationally and by poverty groups over time.

Nutrient	1991		2000		2010		Change between 1991–2010	
	Mean	SE	Mean	SE	Mean	SE	unit/AME/day	%
**National**								
Energy (kJ)	218	8.0	210‡	5.7	302*	6.4	+85†	+39
Protein (g)	7.2	0.3	7.7‡	0.2	9.7*	0.2	+2.5†	+35
Fat (g)	2.5	0.1	2.1*	0.1	3.7*	0.1	+1.2†	+48
Iron (mg)	1.9	0.1	1.7**	0.1	1.6‡	0.0	-0.3†	-15
Zinc (mg)	1.0	0.0	0.9	0.0	1.0**	0.0	-0.0§	-2
Calcium (mg)	324	12.4	286*	7.5	279	5.7	-45†	-14
Vitamin A (μg RAE)	47.3	3.5	46.9‡	2.3	47.8	1.7	+0.6	+1
Vitamin B 12 (μg)	1.3	0.0	1.1*	0.0	1.3*	0.0	-0.0¶	-1
**Extreme poor**								
Energy (kJ)	137	7.2	142‡	5.1	169*	5.0	+31†	+23
Protein (g)	4.7	0.2	5.4**	0.2	5.7‡	0.2	+1.1†	+23
Fat (g)	1.5	0.1	1.4	0.1	2.0*	0.1	+0.4†	+28
Iron (mg)	1.4	0.1	1.3	0.0	1.1*	0.0	-0.3†	-24
Zinc (mg)	0.7	0.0	0.7	0.0	0.7	0.0	-0.1	-9
Calcium (mg)	226	12.3	210	7.0	180*	5.2	-46†	-20
Vitamin A (ug RAE)	36.3	4.6	35.8¶	2.2	35.3‡	2.1	-1.1	-3
Vitamin B 12 (ug)	0.8	0.1	0.7	0.0	0.7	0.0	-0.1	-12
**Moderate poor**								
Energy (kJ)	210	8.7	198	7.7	234*	6.4	+24††	+11
Protein (g)	7.1	0.3	7.3	0.3	7.6‡	0.2	+0.5§	+7
Fat (g)	2.3	0.1	2.0**	0.1	2.9*	0.1	+0.5†	+22
Iron (mg)	1.9	0.1	1.8	0.1	1.3*	0.0	-0.6†	-32
Zinc (mg)	1.0	0.0	0.9‡	0.0	0.8*	0.0	-0.2†	-21
Calcium (mg)	325	14.7	291	11.5	221*	5.9	-104†	-32
Vitamin A (μg RAE)	47.3	3.7	48.2	3.5	37.7**	2.0	-9.6††	-20
Vitamin B 12 (μg)	1.2	0.1	1.0*	0.0	0.9	0.0	-0.3†	-26
**Non-poor**								
Energy (kJ)	293	10.6	261**#	6.7	347*	6.9	+54†	+18
Protein (g)	9.5	0.3	9.5‡	0.2	11.1*	0.2	+1.5†	+16
Fat (g)	3.4	0.1	2.7*	0.1	4.3*	0.1	+0.8†	+25
Iron (mg)	2.4	0.1	2.0*	0.1	1.8**	0.0	-0.6†	-24
Zinc (mg)	1.3	0.0	1.1*	0.0	1.1‡	0.0	-0.2†	-13
Calcium (mg)	411	15.9	337*	8.7	313**	6.4	-97.7†	-24
Vitamin A (ug RAE)	56.8	4.3	54.3	3.0	52.6	1.9	-4.2	-7
Vitamin B 12 (ug)	1.7	0.1	1.4*	0.0	1.5‡	0.0	-0.2†	-13

SE, standard error; AME, adult male equivalent.

Means adjusted for clustering and sample weights. Significance of sensitivity analysis only noted when different from main analysis.

for vitamin A, n = 25,254; for vitamin B12, n = 24,960; for all other nutrients, n = 25,425 households (see methods for explanation)

* Significantly different from previous survey year at P<0.01.

**Significantly different from previous survey year at P<0.05.

† 2010 mean is significantly different from 1991 mean at P<0.01.

†† 2010 mean is significantly different from 1991 mean at P<0.05.

‡ In sensitivity analysis, median intake was significantly different than median intake in previous year at P<0.05.

§ In sensitivity analysis, median intake in 2010 was significantly different than median intake in 1991 at P<0.05.

¶ In sensitivity analysis, a significant change in medians was observed in the opposite direction to that detected by means at P<0.05.

# In sensitivity analysis, no significant change was detected.

When comparing changes among poverty groups, the moderate and non-poor experienced smaller increases in energy, protein and fat, compared to extreme poor households, which are consistent with lower proportional increases in total fish consumption among the moderate and non-poor (Table 2). Moderate and non-poor households show larger proportional decreases in zinc, calcium, vitamin A and vitamin B12 over time compared to extreme poor households. This could be a result of larger proportional decreases in consumption of non-farmed fish by these groups (Table 2). Absolute intakes of nutrients from fish by extreme-poor households are far lower than moderate and non-poor households in each survey year, which is consistent with lower intakes of total fish (Table 2).

## Discussion

Fish consumption has significantly increased from 1991 to 2010; rapid growth in aquaculture has more than compensated, in terms of quantity, for a decline in availability of fish from capture fisheries. This is broadly consistent with increased fish production figures over this period [36], and the general socio-demographic trend with households moving out of poverty, and consumption of higher market value foods (including ASFs) increasing.

However, growth in aquaculture has not sufficiently compensated for the decline in capture fisheries in terms of nutritional quality. Changes in nutrient intakes from fish would be expected to be similar to overall changes in consumption, if nutritional quality of the fish in supply was maintained over time (i.e. a national increase of approximately 30% for all nutrients between 1991 and 2000). This was observed in the increases in animal protein, fat and energy intakes from fish. However, despite an increase in quantity of fish consumed, results show decreased intakes of iron and calcium from fish; and no change in intakes of zinc, vitamin A and vitamin B12 between 1991 and 2010 (results were robust to sensitivity analysis).

Based on our analysis, the only likely explanation for this finding is lower overall nutritional quality of the fish species being consumed in 2010 compared to 1991, related to a greater proportional contribution of farmed fish over time. This is supported by research that has demonstrated the higher nutritional quality of non-farmed species compared to commonly farmed species, with regards to certain micronutrients, including iron, zinc, calcium, vitamin A and vitamin B12 [8, 16].

Given the importance of fish in diets, this reduction in nutritional quality is likely to have exacerbated existing widespread micronutrient deficiencies. The most recent estimates from Bangladesh in 2011–12 show that among non-pregnant, non-lactating (NPNL) women, 57% were zinc deficient, 22% were vitamin B12 deficient, 7.1% were iron deficient, and 5.4% were vitamin A deficient [4]. If nutritional quality of the fish supply in 1991 had been maintained, and consumption was still able to grow to 2010 levels, the average NPNL woman would be consuming an additional 21% of the recommended nutrient intake (RNI) for vitamin B12, 17% of the RNI for calcium, 4% of the RNI for zinc, and 3% of the RNI for iron [37]. Given that NPNL women on average, meet only 25% of the daily iron RNI, and 51% of the daily zinc RNI, this reflects an important contribution.

It could be reasoned that declines in nutrient intakes from a particular food source are of little concern if they can be met from other foods in the diet. The HIES reports published elsewhere show that consumption of ASFs excluding fish increased by 13 g/capita/day from 1991–2010 [29–31], mostly from increases in poultry, potentially compensating to some extent for the reductions in certain micronutrient intakes from fish. This cannot be said for the poor–given that in 2010, more than 80% of extreme poor households had consumed no poultry in the two weeks’ survey period (compared to <4% who consumed no fish). Other recent work investigating quantities of ASFs consumption in rural Bangladesh found that fish consumption was six times higher than poultry–the second most highly consumed ASF [8]. Furthermore, nutritional profiles of ASFs differ, and therefore cannot be considered nutritionally equivalent. For example, fish is often a rich source of long-chain omega 3 fatty acids (not found, or found in small quantities in other ASFs), which are particularly important for cognitive development in children [15]. Vitamin B12 is an essential nutrient required for brain and nervous system function which cannot be produced by the body and is found almost exclusively in ASFs, of which fish is often the richest source [38]. Calcium is essential for bone development as well as many metabolic processes. Milk and dairy products are often assumed to be the most important dietary sources of calcium, however several species of fish in Bangladesh have a much higher calcium content [16, 33], and with the same high calcium bioavailability as milk [39]. ASFs are also recognised as a rich source of highly bioavailable iron due to the high content of haem iron (compared to non-haem iron). The haem iron content of red meat is approximately 40% (of total iron) and can be up to 70%; recent analysis of SIS in Bangladesh has shown haem iron content as high as 93% [40]. Given the importance of fish in diets in Bangladesh (in terms of quantity, nutritional quality and frequency of consumption), this reduction in nutritional quality in fish supply at population level is undoubtedly of concern. In the context of widespread micronutrient deficiencies, even if the negative impacts of declines in nutrient intakes from fish are partially averted in some population groups by substitution with other ASFs, from a population perspective, this still represents an opportunity forgone to improve overall diets.

These results also highlight that the dominant discourse linking fish to food security which confines the nutritional importance of fish to being a source of animal protein, is inadequate and problematic. Protein content of different fish species varies very little—usually by less than 5% (protein content of most species is around 15–20 g/100 g raw, edible parts) [16]. So increases in production and productivity, regardless of species automatically increase availability of protein (as our results show). This is important, particularly as protein from fish is of high quality. However, our results demonstrate that failure to consider fish as an irreplaceable source of micronutrients occludes the ‘bigger picture’ and can be detrimental for nutrition and health.

These results also emphasise the importance of dietary diversity, not only in terms of food groups, as it is often understood, but also diversity of foods within foods groups (e.g. consumption of a diversity of fish species given their unique nutritional profiles). This point is particularly relevant given the above-mentioned transition in aquatic food systems, away from diverse capture fisheries towards less diverse aquaculture systems. Food production systems which promote diversity should be prioritised. One such example is pond polyculture, in which several large fish species are cultured together with SIS in homestead ponds. The different species fulfil different ecological niches within the system, increasing the productivity and nutritional quality of the system as a whole; encouraging frequent harvesting and consumption of SIS by the household, and sale of large fish for income [41]. Public investments in research on the artificial reproduction of key nutrient-rich SIS and the transfer of this technology to private sector hatcheries must be central to this approach. Similar research and investments have preceded growth in production of all the main fish species farmed commercially [42, 43].

Nutrition-sensitive aquaculture must be a complement to diverse capture fisheries, not a substitute for it. Conserving and rebuilding inland, coastal and marine fish stocks through improved management are also essential. One such example is the stocking of nutrient-rich SIS in community-managed wetlands, which improves nutritional quality of production as well as overall productivity and biodiversity of the system [44]. Achieving wider recognition of the essential contributions that capture fisheries make to food and nutrition security is an important step towards ensuring that capture fisheries attains a higher level of priority in global and national policy initiatives, and that their governance is improved for long-term sustainability.

Other factors that influence the nutritional quality of fish species may provide opportunities to improve the contribution of aquaculture systems to nutrition. For example, in response to increasing pressures on marine fisheries as a source of fish meal used as feed in aquaculture systems, modifications in fish feed composition are being investigated as a means to improve the fatty acid profile of farmed fish [45, 46]. Further research within aquatic agricultural food systems, including consideration of other nutrients of public health concern, and analysis of the bioavailability of nutrients are required. WorldFish, the World Bank and social entrepreneurs have recently launched an initiative for attracting investments from major development partners and the private sector to develop and test food systems approaches for optimizing the contribution of fish to improve nutrition and health, especially in women and young children in the first 1,000 days of life.

## Conclusion

The valuable role of aquaculture in Bangladesh in securing the availability and affordability of fish is unquestionable. If growth in this sector had not occurred, declines in nutrient intakes described here would undoubtedly be much more severe, with far more serious implications for nutrition and health. However, the results presented here highlight unintended negative consequences of policy decisions and agricultural investments which are narrowly focused on maximising production and productivity. In doing so, our results challenge the dominant rhetoric that increases in food supply automatically lead to improvements in diet and nutrition. These findings are of significance to many countries experiencing rapid growth in aquaculture alongside declining quantity and diversity of species from capture fisheries. In this light, whilst the findings are specific to Bangladesh, it is possible that this decline of nutritional quality linked to a shift towards greater farmed fish consumption, is occurring on a global scale. As aquaculture becomes an increasingly important food source for many, it must embrace a nutrition-sensitive approach, by considering how changes in food supply affect nutritional quality of diets. To do so requires greater knowledge of the nutritional value of indigenous foods at species/varietal level, and the contributions these foods make in terms of nutrient intakes and dietary patterns, specific to age and sex groups, as well as to differences in rural/urban locations and geographic regions. Indicators used in the monitoring and evaluation of agricultural interventions must go beyond production and productivity, to also include nutritional quality. If the intrinsically linked issues of poverty, food insecurity and malnutrition are to be truly addressed; and for the SDGs to be achieved, agricultural policies must integrate strategies to mitigate trade-offs across multiple sectors, including (but not limited to) nutrition and health.

## Supporting information

S1 File Supporting information. (DOCX)
