# Supplementary material for: Higher fish but lower micronutrient intakes: Temporal changes in fish consumption from capture fisheries and aquaculture in Bangladesh
Source: PLoS One. 2017 Apr 6;12(4):e0175098. doi: 10.1371/journal.pone.0175098 (PMC5383130; doi:10.1371/journal.pone.0175098)
Supplement: S1 File — (DOCX) [file pone.0175098.s001.docx]

# Supporting Information

## Detailed methods

### Fish consumption

The HES of 1991, and the HIESs of 2000 and 2010 record fish consumption according to 37 and 15 categories, respectively. Some categories represent a single fish species, whereas others include several. It was considered important to de-cluster the categories as accurately as possible given the wide differences in nutrient composition of different species. As far as possible, this was done using official production figures from the Department of Fisheries in order to estimate consumption of individual species [36, 47-49]. In cases of insufficient available production data, consumption was estimated to be distributed evenly among species within a category. Individual fish species were then categorised according to predominant source, either capture fisheries or aquaculture, for each survey. Fish that are commonly from both non-farmed and farmed sectors were attributed proportionately between sectors. Official production data were complemented by expert opinion from fisheries and aquaculture specialists in Bangladesh in order to estimate the contribution of fish from the different production sectors as accurately as possible (SI Table 1). The 2010 HIES collected information on foods consumed outside the home, including fish. As this information was not collected in the earlier surveys and accounted for <1% of total fish consumption, this fish category was excluded from analysis.

**Table A. Survey food categories, production weighting, source of production and nutrient composition of fish species.**

| **Survey food code** | **Survey food description** | **Scientific name of species used for nutrient composition** | **Weighting within survey category** | **Source of production** | | **Edible part coefficient** | **Nutrient composition (per 100 g raw, edible parts)** | | | | | | | |
| --- | --- | --- | --- | --- | --- | --- | --- | --- | --- | --- | --- | --- | --- | --- |
|  |  |  |  | **Non-farmed** | **Farmed** |  | **Energy (kJ)** | **Protein (g)** | **Fat (g)** | **Iron (mg)** | **Zinc (mg)** | **Calcium (mg)** | **Vitamin A (µg RAE)** | **Vitamin B12 (µg)** |
| **1991** |  |  |  |  |  |  |  |  |  |  |  |  |  |  |
| 28 | Hilsa | *Tenualosa ilisha* | 100 | 100 |  | 0.83 | 1020 | 16.4 | 18.3 | 1.90 | 1.20 | 220 | 20 | 2.30 |
| 29 | Koi/Magur/Shing/Khalisha | Average* | 100 | 100 |  | 0.867 | 448 | 16.6 | 4.6 | 2.09 | 1.19 | 476 | 100 | 6.39 |
| 30 | Sol | *Channa striatus* | 33.33 | 100 |  | 0.83 | 310 | 18.7 | 0.3 | 0.41 | 0.73 | 96 | 0 | 1.20 |
|  | Gazar | *Channa marulius* | 33.33 | 100 |  | 0.83 | 286 | 17.1 | 0.3 | 0.43 | 0.60 | 9 | 0 | 0.55 |
|  | Taki | *Channa punctatus* | 33.33 | 100 |  | 0.867 | 306 | 18.3 | 0.6 | 1.80 | 1.50 | 766 | 139 | 1.60 |
| 31 | Puti/Sharputi | Average† | 100 | 100 |  | 0.867 | 552 | 16.2 | 7.4 | 2.07 | 2.48 | 916 | 25 | 3.58 |
| 32 | Mala/Kachi/Chala/Chapila | Average‡ | 100 | 100 |  | 0.867 | 362 | 15.0 | 3.2 | 4.24 | 3.28 | 848 | 697 | 6.04 |
| 33 | Chingri | *Macrobrachium malcolmsonii* | 100 | 100 |  | 0.867 | 364 | 15.7 | 2.2 | 13.00 | 3.30 | 1200 | - | - |
| 34 | Dried fish | Average§ | 100 | 100 |  | 0.867 | 1610 | 62.0 | 14.7 | 25.00 | 12.00 | 2540 | 8 | - |
| 35 | Tengra | *Mystus vittatus* | 50 | 100 |  | 0.867 | 428 | 15.1 | 4.6 | 4.00 | 3.10 | 1093 | 12 | 3.50 |
|  | Baim | *Mastacembelus armatus* | 50 | 100 |  | 0.867 | 381 | 17.9 | 1.7 | 1.90 | 1.10 | 449 | 27 | 1.72 |
| 36 | Seafish | Average¶ | 100 | 100 |  | 0.83 | 443 | 17.7 | 4.1 | 1.41 | 1.52 | 745 | 5 | 1.63 |
| 37 | Chital | *Chitala chitala* | 100 | 100 |  | 0.83 | 405 | 17.8 | 2.8 | 1.60 | 0.61 | 104 | 30 | - |
| 38 | Rui | *Labeo rohita* | 45.17 | 30 | 70 | 0.83 | 422 | 18.2 | 3.0 | 0.98 | 1.00 | 51 | 13 | 5.05 |
|  | Katal | *Catla catla* | 32.67 | 30 | 70 | 0.83 | 267 | 14.9 | 0.7 | 0.83 | 1.10 | 210 | 22 | 1.30 |
|  | Mrigel | *Cirrhinus mrigala* | 22.16 | 50 | 50 | 0.83 | 363 | 18.9 | 1.1 | 2.50 | 1.50 | 960 | 15 | 5.57 |
| 39 | Aier | *Sperata aor* | 100 | 100 |  | 0.867 | 373 | 17.0 | 2.3 | 0.90 | 0.23 | 11 | - | - |
| 40 | Pangas | *Pangasianodon hypophthalmus* | 100 | 100 |  | 0.83 | 925 | 16.0 | 17.7 | 0.69 | 0.65 | 9 | 31 | 1.50 |
| 41 | Vetki | Lates calcarifer | 100 | 100 |  | 0.83 | 406 | 18.6 | 2.5 | 1.00 | 0.16 | 24 | 8 | - |
| 42 | Baila | *Glossogobius giuris* | 100 | 100 |  | 0.867 | 292 | 16.6 | 0.4 | 2.30 | 2.10 | 790 | 18 | 2.10 |
| 43 | Mani | *Nandus nandus* | 100 | 100 |  | 0.867 | 338 | 16.7 | 1.7 | 0.84 | 1.60 | 1300 | 60 | 0.90 |
| 44 | Pabda | *Ompok pabda* | 100 | 100 |  | 0.867 | 619 | 16.2 | 9.5 | 0.46 | 0.90 | 91 | - | - |
| 45 | Kakra | *Scylla serrata* | 100 | 100 |  | 0.83 | 411 | 17.9 | 2.9 | 2.60 | - | 183 | 218 | - |
| 46 | Bata | *Labeo bata* | 100 | 100 |  | 0.83 | 446 | 15.9 | 4.7 | 1.20 | 0.94 | 493 | - | - |
| 47 | Tapshi | *Eleutheronema tetradactylum* | 100 | 100 |  | 0.83 | 425 | 20.6 | 2.2 | 0.60 | 0.90 | 37 | 0 | 0.85 |
| 48 | Farsha | *Setipinna phasa* | 100 | 100 |  | 0.867 | 441 | 17.7 | 3.8 | 1.80 | 3.20 | 452 | 12 | - |
| 49 | Khaira | *Gudusia chapra* | 100 | 100 |  | 0.867 | 385 | 15.5 | 3.8 | 7.60 | 2.10 | 1063 | 73 | 6.99 |
| 50 | Dagra†† | *Glossogobius giuris* | 100 | 100 |  | 0.867 | 292 | 16.6 | 0.4 | 2.30 | 2.10 | 790 | 18 | 2.10 |
| 51 | Chanda | *Pseudambassis ranga* | 100 | 100 |  | 0.867 | 400 | 15.5 | 3.8 | 2.10 | 2.60 | 1153 | 336 | 6.42 |
| 52 | Champa | *Scomberomorus commerson* | 100 | 100 |  | 0.83 | 470 | 19.8 | 3.6 | 2.00 | 0.40 | 92 | 30 | - |
| 53 | Kazeli†† | *Ailia coila* | 100 | 100 |  | 0.867 | 751 | 17.1 | 12.6 | 0.82 | 1.20 | 110 | 37 | 4.10 |
| 54 | Laitta | *Channa punctatus* | 100 | 100 |  | 0.867 | 306 | 18.3 | 0.6 | 1.80 | 1.50 | 766 | 139 | 1.60 |
| 55 | Lona hilsa | Tenualosa ilisha | 100 | 100 |  | 0.83 | 1020 | 16.4 | 18.3 | 1.90 | 1.20 | 220 | 0 | 2.30 |
| 56 | Rita†† | *Mystus cavasius* | 100 | 100 |  | 0.83 | 479 | 16.8 | 5.1 | 1.80 | 1.30 | 120 | - | - |
| 57 | Shuri†† | Average¶ | 100 | 100 |  | 0.867 | 443 | 17.7 | 4.1 | 1.41 | 1.52 | 745 | 4 | 1.30 |
| 58 | Boyal | *Wallago attu* | 100 | 100 |  | 0.83 | 339 | 15.4 | 2.1 | 0.80 | 0.27 | 83 | 1 | - |
| 59 | Kalibaus | *Labeo calbasu* | 100 | 50 | 50 | 0.83 | 400 | 17.0 | 3.0 | 1.10 | 0.36 | 13 | - | - |
| 60 | African rui | *Clarias batrachus* | 100 |  | 100 | 0.867 | 326 | 16.5 | 1.3 | 1.20 | 0.74 | 59 | 25 | 4.83 |
| 61 | Silver cup | *Hypophthalmichthys molitrix* | 100 |  | 100 | 0.83 | 435 | 17.2 | 4.1 | 4.40 | 1.40 | 903 | 0 | 0.55 |
| 62 | Talapia | *Oreochromis niloticus* | 100 |  | 100 | 0.83 | 390 | 19.5 | 2.0 | 4.40 | 1.40 | 903 | 10 | 0.70 |
| 63 | Nilotika | *Oreochromis niloticus* | 100 |  | 100 | 0.83 | 390 | 19.5 | 2.0 | 4.40 | 1.40 | 903 | 10 | 0.70 |
| 64 | Others | Average# | 100 | 100 |  | 0.867 | 444 | 16.9 | 4.2 | 2.33 | 2.19 | 713 | 151 | 3.83 |
| **2000** |  |  |  |  |  |  |  |  |  |  |  |  |  |  |
| 41 | Hilsha | *Tenualosa ilisha* | 100 | 100 |  | 0.83 | 1020 | 16.4 | 18.3 | 1.90 | 1.20 | 220 | 20 | 2.30 |
| 42 | Rui | *Labeo rohita* | 39.36 |  | 100 | 0.83 | 422 | 18.2 | 3.0 | 0.98 | 1.00 | 51 | 13 | 5.05 |
|  | Catla | *Catla catla* | 32.79 |  | 100 | 0.83 | 267 | 14.9 | 0.7 | 0.83 | 1.10 | 210 | 22 | 1.30 |
|  | Mrigel | *Cirrhinus mrigala* | 26.48 |  | 100 | 0.83 | 363 | 18.9 | 1.1 | 2.50 | 1.50 | 960 | 15 | 5.57 |
|  | Kal baush | *Labeo calbasu* | 1.37 | 30 | 70 | 0.83 | 400 | 17.0 | 3.0 | 1.10 | 0.36 | 13 | - | - |
| 43 | Boal | *Wallago attu* | 15.65 | 100 |  | 0.83 | 339 | 15.4 | 2.1 | 0.80 | 0.27 | 83 | 1 | - |
|  | Air | *Sperata aor* | 6.81 | 100 |  | 0.83 | 373 | 17.0 | 2.3 | 0.90 | 0.23 | 11 | - | - |
|  | Pangas | *Pangasianodon hypophthalmus* | 77.54 | 100 |  | 0.83 | 925 | 16.0 | 17.7 | 0.69 | 0.65 | 8.6 | 31 | 1.50 |
| 44 | Khalisha/Magur/Shinghi | Average║ | 100 | 100 |  | 0.867 | 351 | 16.93 | 1.9 | 2.5 | 1.38 | 606 | 34 | 7.73 |
| 45 | Koi | *Anabas testudineus* | 100 | 100 |  | 0.867 | 737 | 15.5 | 12.8 | 0.87 | 0.60 | 85 | 295 | 2.38 |
| 46 | Silver carp | *Hypophthalmichthys molitrix* | 84.38 |  | 100 | 0.83 | 435 | 17.2 | 4.1 | 4.40 | 1.40 | 903 | 0 | 0.55 |
|  | Grass carp | *Ctenopharyngodon idella* | 7.72 |  | 100 | 0.83 | 341 | 15.2 | 1.1 | 0.46 | 0.91 | 54 | - | - |
|  | Miror carp | *Cyprinus carpio* | 7.9 |  | 100 | 0.83 | 381 | 16.4 | 2.9 | 1.10 | 2.20 | 37 | 2 | - |
| 47 | Shoal | *Channa striatus* | 33.33 | 100 |  | 0.83 | 310 | 18.7 | 0.3 | 0.41 | 0.73 | 96 | 0 | 1.20 |
|  | Gajar | *Channa marulius* | 33.33 | 100 |  | 0.83 | 286 | 17.1 | 0.3 | 0.43 | 0.60 | 9.3 | 0 | 0.55 |
|  | Taki | *Channa punctatus* | 33.33 | 100 |  | 0.867 | 306 | 18.3 | 0.6 | 1.80 | 1.50 | 766 | 139 | 1.60 |
| 48 | Puti/Big puti | Average† | 50 | 100 |  | 0.867 | 552 | 16.2 | 7.4 | 2.07 | 2.48 | 916 | 25 | 3.58 |
|  | Tilapia/Nilotica | *Oreochromis niloticus* | 50 |  | 100 | 0.83 | 390 | 19.5 | 2.0 | 1.10 | 1.20 | 95 | 10 | 0.70 |
| 49 | Mala/Kachi/Chala/Chapila | Average‡ | 100 | 100 |  | 0.867 | 362 | 15.0 | 3.2 | 4.24 | 3.28 | 848 | 697 | 6.04 |
| 51 | Shrimp | *Macrobrachium malcolmsonii* | 100 | 100 |  | 0.867 | 364 | 15.7 | 2.2 | 13.00 | 3.30 | 1200 | - | - |
| 52 | Dried fish | Average§ | 100 | 100 |  | 0.867 | 1610 | 62.0 | 14.7 | 25.00 | 12.00 | 2540 | 8 | - |
| 53 | Tangra/Eel fish | Average** | 100 | 100 |  | 0.867 | 411 | 16.5 | 3.6 | 3.35 | 2.20 | 792 | 45 | 2.99 |
| 54 | Seafish | Average¶ | 100 | 100 |  | 0.867 | 443 | 17.7 | 4.1 | 1.41 | 1.52 | 745 | 5 | 1.63 |
| 55 | Baila | *Glossogobius giuris* | 50 | 100 |  | 0.867 | 292 | 16.6 | 0.4 | 2.30 | 2.10 | 790 | 18 | 2.10 |
|  | Tapashi†† | *Eleutheronema tetradactylum* | 50 | 100 |  | 0.83 | 425 | 20.6 | 2.2 | 0.60 | 0.90 | 37 | 0 | 0.85 |
| 56 | Others | Average# | 100 | 100 |  | 0.867 | 444 | 16.9 | 4.2 | 2.33 | 2.19 | 713 | 151 | 3.83 |
| **2010** |  |  |  |  |  |  |  |  |  |  |  |  |  |  |
| 41 | Hilsha | *Tenualosa ilisha* | 100 | 100 |  | 0.83 | 1020 | 16.4 | 18.3 | 1.90 | 1.20 | 220 | 20 | 2.30 |
| 42 | Rui | *Labeo rohita* | 40.33 |  | 100 | 0.83 | 422 | 18.2 | 3.0 | 0.98 | 1.00 | 51 | 13 | 5.05 |
|  | Catla | *Catla catla* | 31.41 |  | 100 | 0.83 | 267 | 14.9 | 0.7 | 0.83 | 1.10 | 210 | 22 | 1.30 |
|  | Mrigel | *Cirrhinus mrigala* | 22.80 |  | 100 | 0.83 | 363 | 18.9 | 1.1 | 2.50 | 1.50 | 960 | 15 | 5.57 |
|  | Kal baush | *Labeo calbasu* | 5.46 |  | 100 | 0.83 | 400 | 17.0 | 3.0 | 1.10 | 0.36 | 13 | - | - |
| 43 | Boal | *Wallago attu* | 3.00 | 100 |  | 0.83 | 339 | 15.4 | 2.1 | 0.80 | 0.27 | 83 | 1 | - |
|  | Air | *Sperata aor* | 1.35 | 100 |  | 0.83 | 373 | 17.0 | 2.3 | 0.90 | 0.23 | 11 | - | - |
|  | Pangas | *Pangasianodon hypophthalmus* | 95.65 |  | 100 | 0.83 | 925 | 16.0 | 17.7 | 0.69 | 0.65 | 8.6 | 31 | 1.50 |
| 44 | Khalisha | *Colisa fasciata* | 33.33 | 100 |  | 0.867 | 354 | 15.2 | 2.5 | 4.10 | 2.30 | 1700 | 46 | 5.55 |
|  | Magur | *Clarias batrachus* | 33.33 | 50 | 50 | 0.867 | 326 | 16.5 | 1.3 | 1.20 | 0.74 | 59 | 25 | 4.83 |
|  | Shinghi | *Heteropneustes fossilis* | 33.33 | 50 | 50 | 0.867 | 374 | 19.1 | 1.9 | 2.20 | 1.10 | 60 | 32 | 12.80 |
| 45 | Koi | *Anabas testudineus* | 100 |  | 100 | 0.867 | 737 | 15.5 | 12.8 | 0.87 | 0.60 | 85 | 295 | 2.38 |
| 46 | Silver carp | *Hypophthalmichthys molitrix* | 61.94 |  | 100 | 0.83 | 435 | 17.2 | 4.1 | 4.40 | 1.40 | 903 | 0 | 0.55 |
|  | Grass carp | *Ctenopharyngodon idella* | 10.19 |  | 100 | 0.83 | 341 | 15.2 | 1.1 | 0.46 | 0.91 | 54 | - | - |
|  | Miror carp | *Cyprinus carpio* | 27.87 |  | 100 | 0.83 | 381 | 16.4 | 2.9 | 1.10 | 2.20 | 37 | 2 | - |
| 47 | Shoal | *Channa striatus* | 33.33 | 100 |  | 0.83 | 310 | 18.7 | 0.3 | 0.41 | 0.73 | 96 | 0 | 1.20 |
|  | Gajar | *Channa marulius* | 33.33 | 100 |  | 0.83 | 286 | 17.1 | 0.3 | 0.43 | 0.60 | 9.3 | 0 | 0.55 |
|  | Taki | *Channa punctatus* | 33.33 | 100 |  | 0.867 | 306 | 18.3 | 0.6 | 1.80 | 1.50 | 766 | 139 | 1.60 |
| 48 | Puti/Big puti | Average† | 50 | 100 |  | 0.867 | 552 | 16.2 | 7.4 | 2.07 | 2.48 | 916 | 25 | 3.58 |
|  | Tilapia/Nilotica | *Oreochromis niloticus* | 50 | 100 | 100 | 0.83 | 390 | 19.5 | 2.0 | 1.10 | 1.20 | 95 | 10 | 0.70 |
| 49 | Mala/Kachi/Chala/Chapila | Average‡ | 100 | 100 |  | 0.867 | 362 | 15.0 | 3.2 | 4.24 | 3.28 | 848 | 697 | 6.04 |
| 51 | Shrimp | *Macrobrachium malcolmsonii* | 100 | 100 |  | 0.867 | 364 | 15.7 | 2.2 | 13.00 | 3.30 | 1200 | - | - |
| 52 | Dried fish | Average§ | 100 | 100 |  | 0.867 | 1610 | 62.0 | 14.7 | 25.00 | 12.00 | 2540 | 8 | - |
| 53 | Tangra/Eel fish | Average** | 100 | 100 |  | 0.867 | 411 | 16.5 | 3.6 | 3.35 | 2.20 | 792 | 45 | 2.99 |
| 54 | Seafish | Average¶ | 100 | 100 |  | 0.867 | 443 | 17.7 | 4.1 | 1.41 | 1.52 | 745 | 5 | 1.63 |
| 55 | Baila | *Glossogobius giuris* | 50 | 100 |  | 0.867 | 292 | 16.6 | 0.4 | 2.30 | 2.10 | 790 | 18 | 2.10 |
|  | Tapashi†† | *Eleutheronema tetradactylum* | 50 | 100 |  | 0.83 | 425 | 20.6 | 2.2 | 0.60 | 0.90 | 37 | 0 | 0.85 |
| 56 | Others | Average# | 100 | 100 |  | 0.867 | 444 | 16.9 | 4.2 | 2.33 | 2.19 | 713 | 151 | 3.83 |

* Average of *Colisa fasciata, Heteropneustes fossilis, Clarias batrachus, Anabas testudineus*.

† Average of *Puntius ticto, Puntius sophore, Puntius sarana*.

‡ Average of *Amblypharyngodon mola, Gudusia chapra, Chela cachius, Corica soborna*.

§ Average of *Puntius sophore, Setipinna phasa*.

¶ Average of *Liza parsia, Johnius argentatus, Sillaginopsis panijus, Stolephorus tri, Pampus argenteus*.

# Average of *Mastacembelus armatus, Esomus danricus, Osteobrama cotio cotio, Hyporhamphus limbatus, Notopterus notopterus, Mystus cavasius, Lepidocephalichthys guntea, Ailia coila, Xenontedon cancila, Eleotris fusca, Nandus nandus, Ompok pabda, Botia dario, Macrognathus aculeatus.*

║ Average of *Colisa fasciata, Heteropneustes fossilis, Clarias batrachus*.

** Average of *Mystus vittatus, Mastacembelus pancalus*.

†† No nutrition composition data available for fish species, so data of similar species, or species from same family used.

### Use of the adult male equivalent

Food consumption from household surveys is normally reported as per capita means; total household food consumption is divided by the number of people present in the household (children and adults are treated equally). This is a limitation because it does not take into account the different physiological energy requirements of different age and gender groups [32]. An alternative method is to divide household consumption according to the number of AMEs in the household, which are estimated from energy requirements of individual household members, based on age and gender, as a proportion of the energy requirement of an adult male (SI Table 2) [32]. This method produces a more accurate estimate of the adequacy of household food consumption. The energy requirements (ERs) for different age and gender groups were estimated based on FAO/WHO methodology [50], assuming a moderate physical activity level (PAL). This calculation requires details of individual body weight which were not available from the surveys, and so alternative methods for different age groups were applied, as explained below. For children under ten years, weight data from WHO growth reference data were used [51, 52]. There are no universal growth charts for child weight over the age of nine years, so the 2007 WHO growth reference for body mass index (BMI) for age, and height for age, with adjustments for the short stature of Bangladeshis, were used. This adjustment is important because adult height cannot be assumed to be equal across different populations, unlike the universal child growth standards. The maximal heights for men and women were adjusted to heights observed in Bangladesh in the 2011 Bangladesh Demographic and Health Survey (BDHS), and the intermediate measures were smoothed by reducing the growth pattern to match the maximal height using the following formula:

$$x_{a,s}=\left( \left( h_{a, s}- h_{a120,s} \right) \times\frac{m_{s}}{h_{a228,s}} \right)+h_{a120,s}$$

Where

$x_{a,s}=$Adjusted height of an individual of a given age and sex

$h_{a,s}=$ Height of an individual from the 2007 WHO growth reference curve of the given age and sex

$h_{a120,s}=$ Height of an individual from the 2007 WHO growth reference curve of the given sex at age 120 months

$m_{s}=$Mean height of an adult individual of the given sex as observed in the population

$h_{a228,s}=$Adult height of an individual from the 2007 WHO growth reference curve of the given sex at age 228 months

These heights were then used to impute weights, using the mean 2007 WHO growth reference BMI for age (by age in months). Using the formulae provided by the FAO manual, daily ERs for adolescents of each month of age and sex were calculated, then averaged over the year. To obtain estimates of adult weight, we used the mean BMI of adults of 19 years from the 2007 WHO growth curves (female BMI=21.427; male BMI= 22.19) and the mean adult heights observed in Bangladesh. These two measures were used to impute an optimal adult weight (female, 48.84 kg; male, 58.38 kg), and combined with a PAL of 1.85 (moderately active) to estimate ERs for adults.

The AME method can be further improved by accounting for increased energy needs of pregnant or lactating women within each household. No data were available within the surveys on pregnancy or lactation status of women, however, given that breastfeeding rates are relatively high in Bangladesh (90% of children until age two years in 2011 [53] and 81% in the first BDHS in 1993-94 [54]) , it was assumed that for every household with a child two years of age or less that they would be breastfed, and so the household AME was increased to account for this. The increased energy requirement for breastfeeding was estimated at 20% of the energy cost of milk production (as milk produced is converted to food energy to meet the needs of the infant with 80% efficiency). This is highest in the first six months (2.8 MJ/day) and decreases to (1.925 MJ/day) thereafter [50], so for households with an infant less than one year old, the energy cost of lactation was averaged over the first 12 months (((2.8+1.925)/2) X 0.8 efficiency), and for households with an infant aged one year, it was estimated as 1.925 MJ/day X 0.8 efficiency.

**Table B. Adult male equivalent values for age and gender groups in Bangladesh.**

| **Age (year)** | **Females** | **Males** |
| --- | --- | --- |
| <1 | 0.20 | 0.22 |
| 1.0–1.9 | 0.29 | 0.32 |
| 2.0–2.9 | 0.36 | 0.39 |
| 3.0–3.9 | 0.40 | 0.43 |
| 4.0–4.9 | 0.44 | 0.47 |
| 5.0–5.9 | 0.47 | 0.51 |
| 6.0–6.9 | 0.51 | 0.55 |
| 7.0–7.9 | 0.55 | 0.59 |
| 8.0–8.9 | 0.59 | 0.64 |
| 9.0–9.9 | 0.64 | 0.69 |
| 10.0–10.9 | 0.67 | 0.71 |
| 11.0–11.9 | 0.72 | 0.76 |
| 12.0–12.9 | 0.75 | 0.83 |
| 13.0–13.9 | 0.78 | 0.90 |
| 14.0–14.9 | 0.80 | 0.96 |
| 15.0–15.9 | 0.81 | 1.00 |
| 16.0–16.9 | 0.82 | 1.04 |
| 17.0–17.9 | 0.82 | 1.07 |
| 18.0–18.9 | 0.83 | 1.08 |
| 19.0-29.9 | 0.78 | 1.02 |
| 30.0–59.9 | 0.80 | 1.00 |
| ≥65 | 0.71 | 0.82 |
| Lactation^ (children<1) | 0.04 |  |
| Lactation^ (children 1.0–1.9) | 0.03 |  |

^in addition to base requirement for age group

### Matching household roster and household consumption modules

The age and sex of household members (used to calculate AMEs) is recorded in the household roster module of the survey. However, the presence or absence of household members and guests on each day within the food consumption reference period is not recorded in the survey, posing difficulty in estimating household AMEs that accurately reflect the age and sex distribution of individuals among whom food was distributed. The survey does record the number of females and males under and over age 10 years who are present in the household on each day food consumption was measured, allowing for assumptions to be made as to the presence or absence of household members and guests. In cases where the number of people present for food consumption did not precisely match the household roster information (1991, n= 4366; 2000, n=4809; 2010, n=7284) the household AME was adjusted. Note that these discrepancies do not indicate error in data collection; rather they provide detail on the number of individuals consuming food on specific days within the reference period, which may deviate from the individuals who are considered ‘household members’ for other purposes. If the number of individuals in any category (females and males under and over age 10 years) in the consumption module was *more than* the number recorded in the roster, an average AME (of all individuals within that category in the survey) was added to the household AME. If the number of individuals in any category (females and males under and over age 10 years) in the consumption module was *less than* the number recorded in the roster, an average AME (of all household members within that category) was subtracted from the household AME. In cases where data on the number of people present in the household for food consumption were missing (1991, n=69; 2000, n=43; 2010, n=0), household AMEs were estimated solely on information contained in the household roster information.

## Strengths and limitations

This study has drawn on high quality nationally representative survey data using multiple times points, in combination with comprehensive food composition data at species level, in a unique analysis of changes in nutritional quality of fish consumed over time. The use of AMEs rather than per capita estimates takes into consideration changes in age and sex distributions over time, as well as a more accurate estimate of the adequacy of household food consumption in each survey year. Whilst this is an improvement on per capita estimates, the AME only considers energy requirements and does not account for different physiological requirements of micronutrients by different age and sex groups.

The potential for bias to influence results has been removed or minimised where possible, however, some aspects were unavoidable. Of note are considerable changes to survey methodology implemented in the 2000 HIES onwards (see detailed methods). Data checking at field level was conducted in 2000 and 2010, allowing opportunity for enumerators to re-visit households with extreme or missing values. This means that data from 1991 are more likely to be effected by outlying values, although the sensitivity analyses conducted in this study in the form of quantile regression mitigated this effect. Furthermore, households with unrealistic levels of fish consumption in the local context (>500 g/AME/day) were excluded (n=15), all of which were from the 1991 survey. The questionnaire was also changed in 2000, with the total number of fish categories recorded consolidated. The effect of this consolidation was largely mitigated by re-expanding the fish categories according to production figures as described in Detailed methods section and in SI Table 1; however, the number of fish categories was still less diverse than those recorded in previous years. This was also the first year that the recall period for food consumption was increased (from 24 hours to 2-days), and the number of repeated measures and reference period was decreased (from 30 measurements over a 30 day reference period to 7 measurements over a 14 day reference period). Generally, a higher number of repeated measures of food consumption and a longer reference period increase the reliability of results, however, this must be balanced against increased interviewer and interviewee burden and fatigue. As such, there is no consensus as to the level of improvement expected from seven to 30 measurements and a reference period of 30 instead of 14 days [55]. The increase in recall period in later survey years is likely to underestimate consumption (some consumption may be overlooked over a longer recall period). It should also be emphasised that food consumption recorded in HIESs reflects ‘apparent’ consumption and does not consider food wastage, plate waste and foods consumed outside the home, or consumption patterns among individuals. Other research has shown that large fish species have higher plate waste compared to SIS which are normally consumed whole [56]. Given that farmed fish (which are typically large in size) made up a greater proportion of consumption in later survey years, this may have disproportionately overestimated actual intakes. To minimise the impact of this limitation in the nutrient intake analysis, we adjusted for this by using an edible part conversion for small and large fish before estimating nutrient intakes (as nutrient composition refers to edible parts only). It is also likely that more food is being consumed outside the home in later survey years. By excluding this category, we may have underestimated actual fish consumption in 2010 (this information was not recorded in earlier surveys). Another minor limitation of the study was the absence of food composition data for vitamin B12 and vitamin A for some fish species. A small number of households consuming only fish with these missing data were therefore excluded from analysis of those specific nutrients (vitamin A, n=171; vitamin B12, n=465) to minimise impacting results. However, many households consumed a mix of species (with both known and unknown content of vitamin A or vitamin B12) and so were included in analysis of these nutrients. Assuming that these species of unknown vitamin content are a source of those vitamins, vitamin A and vitamin B12 intakes from fish are likely to have been underestimated.

# Additional references

47. FRSS. Fisheries statistical yearbook of Bangladesh: 1991-1992. Bangladesh: Fisheries Resources Survey System (FRSS), Department of Fisheries, 1993.

48. FRSS. Fisheries statistical yearbook of Bangladesh: 2000-2001. Bangladesh: Fisheries Resources Survey System (FRSS), Department of Fisheries, 2002.

49. Toufique KA, Belton B. Is aquaculture pro-poor? Empirical evidence of impacts on fish consumption in Bangladesh. World Dev. 2014;64:609-20. doi: http://dx.doi.org/10.1016/j.worlddev.2014.06.035.

50. FAO, WHO, UNU. Human energy requirements: report of a joint FAO/WHO/UNU expert consultation. Rome: Food and Agriculture Organization, 2004.

51. WHO. WHO child growth standards. Geneva: World Health Organization, 2006.

52. WHO. Development of WHO growth reference for school aged children and adolescents. Geneva: World Health Organization, 2007.

53. NIPORT, Mitra and Associates, ICF International. Bangladesh demographic and health survey 2011. Dhaka; Maryland: National Institute of Population Research and Training, Mitra and Associates, ICF International, 2013.

54. NIPORT, Mitra and Associates, Macro International Inc. Bangladesh demographic and health survey 1993-94. Dhaka; Maryland: National Institute of Population Research and Training, Mitra and Associates, Macro International Inc, 1994.

55. Smith LC, Dupriez O, Troubat N. Assessment of the Reliability and Relevance of the Food Data Collected in National Household Consumption and Expenditure Surveys. International Household Survey Network, 2014.

56. Roos N. Fish consumption and aquaculture in rural Bangladesh: Nutritional impact and production potential of culturing small indigenous fish species (SIS) in polyculture with commonly cultured carps [Thesis]. Copenhagen, Denmark: The Royal Veterinary and Agricultural University; 2001.
